# Ethylhydrocuprein in Pneumonia

**Published:** 1915-11

**Authors:** 


### ABSTRACTS

**Ethylhydrocuprein in Pneumonia.** Moore, Jour. of Exper. Med., page 269, 1915, finds that this derivative of hydroquinone kills all four types of pneumococcus (classification of Cole, Diches and Gillespie) in vitro, in 18 hours, in dilutions up to a million. Its influence on other bacteria is so much less that it is possible that this fact may be applied as a differential test. As a prophylactic (in mice) protection may be afforded against 1,000 ti es the minimal lethal dose. 56 of 85 mice infected with 100 minimal lethal doses or less, recovered. All controls died. Unfortunately, 13 mice died apparently as the result of poisoning. (Note: The average blood mass of an adult is about 5 liters or 5 million milligrams. While of course, this does not represent the entire volume of tissue involved in an infection nor does the blood necessarily contain the whole of a dose of any drug administered at any given time, we would estimate the human dose at 5 milligrams. This would be expected to be well within the limit of toxic action of any hydroquinone derivative not containing some other ingredient of marked toxic power).

